# The added value of food frequency questionnaire (FFQ) information to estimate the usual food intake based on repeated 24-hour recalls

**DOI:** 10.1186/s13690-017-0214-8

**Published:** 2017-10-30

**Authors:** Cloë Ost, Karin A. A. De Ridder, Jean Tafforeau, Herman Van Oyen

**Affiliations:** 1 Department of Public Health and Surveillance, Unit Surveys, Lifestyle and Chronic Diseases, Scientific Institute of Public Health, Juliette Wytsmanstraat 14, 1050 Brussels, Belgium; 2 Department of Public Health and Surveillance, Scientific Institute of Public Health, Juliette Wytsmanstraat 14, 1050 Brussels, Belgium; 3 Department of Public Health, Ghent University, Ghent, Belgium

**Keywords:** Usual intake, Food frequency questionnaire, FFQ, 24-hour recall, Episodically consumed foods, Statistical modeling methods, Never-consumers, Spade

## Abstract

**Background:**

Statistical methods to model the usual dietary intake of foods in a population generally ignore the additional information on the never-consumers. The objective of this study is to determine the added value of Food Frequency Questionnaire (FFQ) data allowing distinguishing the never-consumers from the non-consumers while modeling the usual intake distribution.

**Methods:**

Three food items with a different proportion of never-consumers were selected from the database of the Belgian food consumption survey of 2004 (*N* = 3200). The usual intake distribution for these food items was modeled with the Statistical Program for Analysis of Dietary Exposure (SPADE) and modeling parameters were extracted. These parameters were used to simulate (a) a new database with two 24-h recalls per respondent and (b) a “true” usual intake distribution. The usual intake distribution from the new database was obtained by modeling the 24-h recalls with SPADE, once without and once with the inclusion of the FFQ data on the never-consumers. Ratios were calculated for the different percentiles of the usual intake distribution: the modeled usual intake (g/day) (for both SPADE with and without the inclusion of FFQ data on never-consumers) was divided by the corresponding percentile of the simulated “true” usual intake (g/day). The closer the ratio is to one, the better the model fits the data.

**Results:**

Inclusion of the FFQ information to identify the never-consumers did not improve the estimation of the higher percentiles of the usual intake distribution. However, taking into account this FFQ information improved the estimation of the lower percentiles of the usual intake distribution even when the proportion of never-consumers was low.

**Conclusions:**

The inclusion of FFQ information to identify the never-consumers is beneficial when interested in the whole usual intake distribution or in the lower percentiles only, no matter how low the proportion of never-consumers for that food item may be. However, when interest is only in the higher percentiles of the usual intake distribution, inclusion of FFQ information to identify the never-consumers will have no benefit.

**Electronic supplementary material:**

The online version of this article (doi:10.1186/s13690-017-0214-8) contains supplementary material, which is available to authorized users.

## Background

Studies comparing dietary and disease patterns in large populations provided evidence for the relation between nutrition and disease incidence. This led to the recognition that an unhealthy diet and lifestyle factors, such as a lack of physical activity, are key risk factors for developing a large variety of chronic conditions, such as cardiovascular diseases, cancer and diabetes [1–3]. This illustrates the importance of assessing the prevalence and distribution of food health indicators in the population.

Information on the diet of a population can be obtained by using a food consumption survey, where the food and nutrient intake can be assessed at an individual level. There is a large variety in dietary collection methods that are available for conducting such surveys. Many of them make use of a (repeated) 24-h recall (24HR), where the respondent is asked to reproduce all the types and amounts of foods consumed during the preceding full day. However, the measurement of the usual food intake is challenging when the number of 24HRs is limited [1, 4–10].

A first shortcoming is that an individual’s food consumption varies from day to day. In addition 24HRs suffer from measurement error, due to recall bias, the use of standard recipe files, etc. These limitations result in a substantial within-individual variability, which leads to a poor estimate of the usual intake distribution [5–9, 11–13]. In practice, the within-individual variability tends to widen the usual intake distribution, which will result in an overestimation of the more extreme percentiles [5, 6, 13]. The majority of the statistical methods consider this first drawback, by integrating out (removing) the within-individual variation from the usual intake distribution during modeling [5–7, 14–17].

The use of a limited number of 24HRs has another drawback, namely it can become very challenging to capture infrequently consumed foods, which makes it difficult to differentiate the non-consumers from the never-consumers [6, 7, 16, 18, 19]. Non-consumers are participants that sometimes consume some specific food items, but did not have consumption on any of the recall days. Never-consumers are participants who do never consume a particular food, nor on any recall day nor on any other day [1, 6, 7, 19]. This second drawback, the difficulty of differentiating the never-consumers from the non-consumers, is generally not considered during the modeling of the usual intake distribution. Also during the analysis of the BNFCS2004 (Belgian National Food Consumption Survey), the available information on the never-consumers was ignored.

A possible solution is to supplement the 24HR data with additional information about the frequency of consumption, such as the one collected with a Food Frequency Questionnaire (FFQ). The latter contains more information on the long term dietary behaviour. This approach allows for the identification of never-consumers of a given food in a population, provided that the FFQ contains a frequency category “never” [1, 6, 7, 16, 18, 19].

The objective of this study is to determine with a simulation study the added value of FFQ information to distinguish the never-consumers from the non-consumers during the modeling of the usual intake distribution. Subsequently we evaluated whether the added value depends on the proportion of never-consumers. Also Goedhart et al. [6] performed a simulation study, where they amongst others assessed the effect of the use of FFQ information to identify the never consumers. However, Goedhart et al. [6] used artificial data to assess the effect, while in this study the simulation will be based on real food items whose intake was assessed in the Belgian population during the BNFCS2004.

## Methods

### Data of the BNFCS 2004 study

Three- thousand two-hundred individuals, who were 15 years or older participated to the BNFCS2004. The goal of the survey was to describe the usual food consumption in Belgium in both genders and in four pre-defined age-groups (15–18 years, 19–59 years, 60–74 years and ≥75 years) separately. The sample size calculation indicated the need for 400 individuals per group. Individuals were selected using a multistage sampling procedure from the national population register [1].

The study design of the BNFCS2004 followed largely the recommendations of the European Food Consumption Survey Method project (EFCOSUM) [4, 10]. A twice repeated non-consecutive face-to-face 24HR and a self-administered FFQ (covering a 12 month period) were used to gather information on food intake. The 24HR was repeated once to obtain more details on the within-individual variation and randomly included (in a large group of individuals) all seasons of the year and all days of the week [1].

EPIC-Soft (European Prospective Investigation into Cancer and Nutrition Software) was used to obtain standardized 24HR interviews [20]; the program was adapted to the Belgian dietary context [1]. The FFQ contained a frequency category “never”, which is essential to make the distinction between (non-)consumers and never-consumers [1]. More detailed information about the study design can be found in De Vriese et al. [1] and on the website of the Scientific Institute of Public Health [2].

### Statistical program to assess dietary exposure

The Statistical Program for the Assessment of Dietary Exposure (SPADE), an R package developed at the Dutch National Institute of Public Health was selected to estimate the usual intake distribution [21, 22], because both R and the SPADE package are freeware. In addition SPADE allows including information on the never-consumers without a large increase in the analysis time [21, 22]. For the data simulation we used R version 3.1.1 and SPADE version spade.rivm_v2.32.12.

SPADE provides different modeling options. This study only made use of the SPADE 2-part model which models episodical (non-daily) intake [21, 22].

**Fig. 1:**
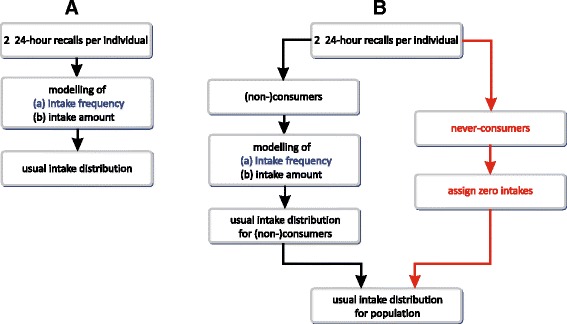
Basic idea of the SPADE 2-part models. Legend: The panel on the left (**a**) shows the SPADE 2-part model without the inclusion of the information on the never-consumers. The panel on the right (**b**) shows the SPADE 2-part model with the inclusion of the information on the never-consumers. Note that based on the FFQ information the population is divided into the consumers and non-consumers on one hand; and the never-consumers on the other hand [21, 22]

The panel on the left in Fig. 1 shows the basic steps of the SPADE 2-part model without inclusion of the never-consumers information: 24HRs of all respondents are used to model (a) the intake frequency and (b) the intake amount. Combining both results in the usual intake distribution for whole the population [21, 22].

The panel on the right in Fig. 1 presents the basics steps of the SPADE 2-part model with inclusion of the never-consumers information. The latter get assigned a zero usual intake. The modeled usual intake distribution of the (non-) consumers and the zero intakes of the never-consumers are combined to obtain the global usual intake, which will reflect the correct proportion of never-consumers [21, 22].

**Fig. 2:**
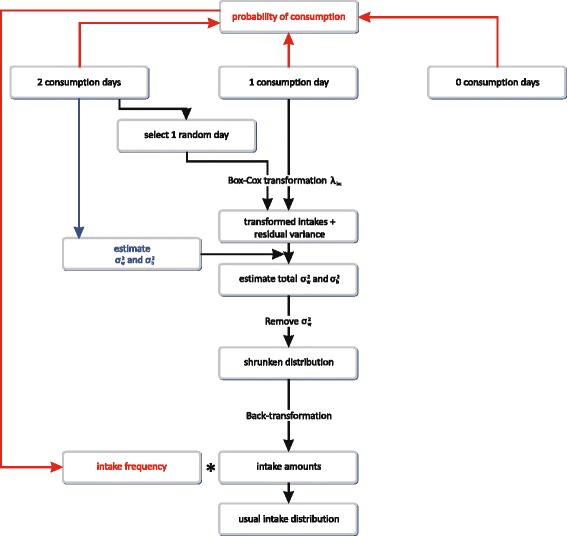
Detail of the intake frequency and the intake amount modeling in the SPADE 2-part model. Legend: Firstly, the SPADE 2-part model estimate the intake frequency and then it estimates the intake amounts. Finally, the intake frequency and the intake amount are combined to obtain the usual intake distribution of (a) whole the population when information on the never-consumers is not taken into account; or (b) the consumers only when information on the never-consumers is taken into account [21, 22]

Figure 2 shows in detail how SPADE models the usual intake distribution. Firstly the consumption frequency is modeled with a beta-binomial model as a function of age. Secondly the consumption amount is modeled. The intake amounts are transformed to normality using a Box-Cox transformation. These transformed amounts are then modeled as a function of age by a fractional polynomial regression and all model parameters are estimated including the total residual variance. The latter has to be partitioned in the between- and within- individual variance. A Gaussian quadrature back-transformation is subsequently used to (a) integrate out the within-individual variance and (b) to back-transform the resulting shrunken distribution to the original scale [21–23].

In the third step the distributions of the intake frequency and intake amount are combined by a Monte Carlo simulation to obtain the usual intake distribution [21, 22].

More detailed information on the SPADE modeling can be found in Additional file 1.

### Selection of the food items

We used the following criteria to select the food items used in the current study: (a) they needed to have different proportions of never-consumers, and (b) even when the proportion of never-consumers was large, the amount of participants consuming the food on both recall days had to be sufficiently large, to avoid convergence problems in SPADE (convergence problems occur when the available amount of data is insufficient to obtain an adequate model fit) [5, 21, 22].

### Data simulation

#### The simulation of a new database

The simulated BNFCS2004 was generated by simulating two 24HRs and basic FFQ information (only information on never-consumers versus consumers). The simulated BNFCS2004 was limited to individuals, aged 15–74 years (*n* = 2363). The simulation was performed stratified in the three different age groups (15–18 years, 19–59 years and 60–74 years), which allows for more variation of the food consumption in function of age. The simulation took place in two stages: (a) simulate the consumers only and (b) simulate the never-consumers only (never-consumers are individuals who indicated in the FFQ that they never consumed the food item during the last 12 months).

##### Simulation of the consumers only – Simulated BNFCS2004 consumers only

For the simulation of the consumers, an approach similar to Souverein et al. [9] was used. SPADE can model both the intake frequency and the intake amounts in function of age [21, 22]. To avoid convergence problems only the intake amounts were modeled in function of age during the simulation.

**Fig. 3:**
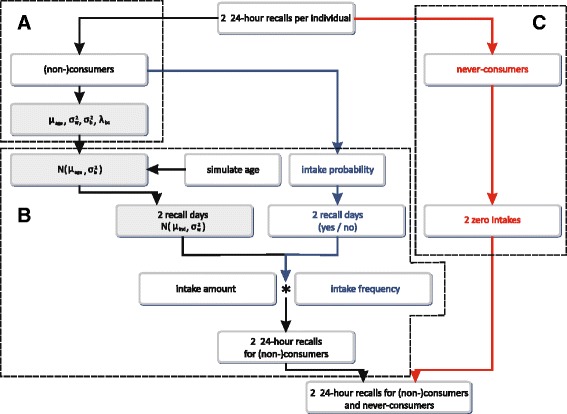
Simulation of the simulated BNFCS2004. Legend: The “simulated BNFCS2004” consisting of two 24HRs per individual was simulated separately in the three age strata: the adolescents (15–18 years), the adults (19–59 years) and the elderly (60–74 years). The grey-shaded steps are performed at the transformed scale. In *box A*, the consumers only in the “original BNFCS2004” are modeled to extract the needed modeling parameters. In *box B*, these parameters were used to simulate two 24 HRs for every (non-) consumer resulting in the “simulated BNFCS2004 for consumers only”. In *box C*, the correct proportion of never-consumers gets assigned two 24 HRs with an intake amount equal to zero. Finally, the two 24 HRs of the (non-) consumers and the never-consumers are combined to obtain the “simulated BNFCS2004”

First all never-consumers were excluded from the original BNFCS2004 database using FFQ data, resulting in a subdatabase with consumers only. Then the SPADE 2-part model without information on the never-consumers was used to obtain the usual intake distribution for consumers only from the original BNFCS2004. During the modeling some parameters were extracted: the mean usual intake for every age (μ_age_), the within-individual standard deviation (σ_w_), the between-individual standard deviation (σ_b_) and the Box-Cox transformation parameter (λ_bc_) (Fig. 3 box A).

After the extraction of all needed parameters the simulation could start on the transformed scale. Firstly the age for all respondents was simulated, making the assumption that the age was uniformly distributed in each of the three age strata. Then each respondents’ mean usual intake was simulated, using a normal distribution, with the mean equal to the age dependent mean usual intake and with the variance equal to the between-individual variance. Next two 24HRs were simulated for each respondent using again a normal distribution with the mean equal to the individuals mean usual intake (simulated in the previous step) and the variance equal to the within-individual variance. The within-individual variance was assumed to be equal for each individual. These intakes were then back-transformed to the original scale using λ_bc_.

During this simulation also the consumption frequency must be considered. This was simulated using a beta-binomial model taking into account the mean intake frequency and the correlation of the intake frequencies (Fig. 3 box B). In a final step the distributions of the intake frequency and the intake amount were combined.

##### Simulation of the never-consumers – Simulated BNFCS2004 never-consumers only

The correct number of never-consumers in each age stratum was calculated based on the FFQ data of the original BNFCS2004. For each never-consumer two 24HRs with a consumption equal to zero were generated resulting in the “simulated BNFCS2004 never-consumers only” (Fig. 3 box C).

#### The simulation of a “true” usual intake distribution

The simulation of the simulated “true” usual intake distribution was very similar and was based on the methods described by Goedhart et al. [6], Tooze et al. [8] and Souverein et al. [9].

##### Simulation of the consumers only – Simulated “true” usual intake distribution consumers only

**Fig. 4:**
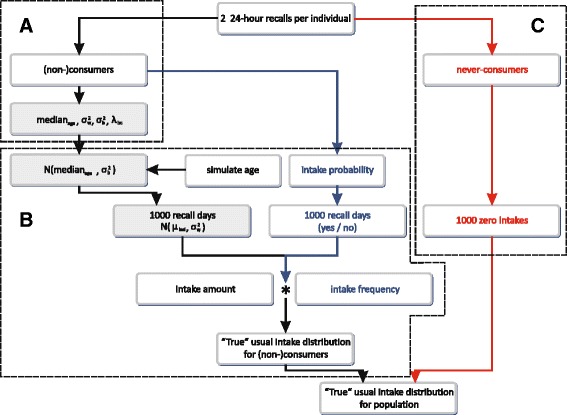
Simulation of the “true” usual intake distribution. Legend: The “simulated BNFCS2004” consisting of two 24-h recalls per individual was simulated separately in the three age strata: the adolescents (15–18 years), the adults (19–59 years) and the elderly (60–74 years). The *grey*-shaded steps are performed at the transformed scale. In *box A*, the needed modeling parameters were extracted by modeling the original BNFCS2004. In *box B*, these parameters were used to simulate one thousand 24 HRs for every consumer. The median intake over these one thousand simulated days results in the “true usual intake distribution for consumers only”. In *box C*, the correct proportion of never-consumers gets assigned one thousand 24 HRs with an intake amount equal to zero, which corresponds to a usual intake of zero. Finally, the usual intake distributions of the consumers and the never-consumers are combined to obtain the “simulated true usual intake distribution”

A “true” usual intake distribution was obtained by simulating 15,000 individuals, similar steps as described above were used. Instead of simulating two 24 HRs per individual, one thousand 24 h were simulated for each individual. The median intake over these thousand days can be considered as the “true” usual intake on a consumption day for that individual, consequently almost no within-individual variance was left. Taking into account the intake frequency will thus directly results in the simulated “true” usual intake distribution, without the need for additional modeling (Fig. 4, box B).

##### Simulation of the never-consumers – Simulated “true” usual intake for never-consumers

The procedure was exactly the same as described for the simulated BNFCS2004 never-consumers only (Fig. 4 box C).

More detailed information on the simulation process can be found in Additional file 1.

### Evaluation of the simulation

Firstly the center (mean and median) of the simulated BNFCS2004 and the simulated “true” usual intake distributions for consumers only must be similar to that of the original BNFCS2004 for consumers only.

Secondly the within-, between-individual and the total residual variance of the simulated BNFCS2004 should be similar to those obtained in the original BNFCS2004. However, in the simulated “true” the variance should be similar to the between-individual variance of the original BNFCS2004.

### Effect of inclusion of FFQ information during modeling

To assess the usual intake distribution for the different food items for the whole Belgian population (15–74 years), the (non-)consumers and never-consumers were combined in all age strata and then all age strata were merged together. In other words the simulated BNFCS2004 was obtained by combining the simulated BNFCS2004 consumers only and the simulated BNFCS2004 never-consumers only. Similarly the simulated “true” usual intake distribution was obtained by merging the simulated “true” usual intake distribution consumers only and the simulated “true” usual intake for never-consumers. Because of the stratified design (by age) of the simulations, normalized survey weights were calculated and used during the analysis with SPADE.

Two versions of the SPADE 2-part model were used to model the simulated BNFCS2004 in order to obtain the usual intake distributions: firstly a model not including the information on the never-consumers: in this situation everyone is considered as a potential consumer. And secondly the model that considered the information on the never-consumers, which allows for taking into account the correct proportion of never-consumers in the population [21, 22]. Based on those models the weighted percentiles of the usual intake distribution were estimated (5; 25; 50; 75 and 95%). The simulated “true” usual intake distribution does not require any modeling. The same percentiles could be determined directly after taking into account the normalized weights.

The difference in the fit of both SPADE models (without and with the inclusion of information on the never-consumers) was evaluated using relative differences. The ratios of the modeled usual intake distributions (obtained from the simulated BNFCS2004) versus the simulated “true” usual intake distribution were calculated. E.g. the usual intake amount (g/day) obtained by one of the SPADE models was divided by the corresponding usual intake amount (g/day) obtained by the simulated “true” usual intake distribution, and this for all percentiles. The closer the ratio is to one, the better the SPADE 2-part model resembles the simulated “true” usual intake distribution at the given percentile.

The relative differences obtained by both models were also plotted in a graph in function of the percentiles. Two specific outcomes with undefined ratios were taken into account: (a) when a ratio of (0 g/day)/(0 g/day) is obtained, which indicates a perfect fit, the ratio will get assigned a value of one, and (b) the ratio (x g/day)/(0 g/day) will get assigned an artificial value of 0.6 to make clear in the graphs that the fit was not perfect.

Goedhart et al. [6] suggested that three replicate simulations are sufficient to check whether replicates are similar. Therefore a sensitivity analysis was done by repeating the simulation three times (four simulations in total) to evaluate the variability of the simulations.

The above described procedure was performed for three different food items (water, cheese and fat spread) with a different proportion of never consumers (respectively 1.9, 6.7 and 31.7%).

## Results

### Selected food items

Three food items, being water, cheese and fat spread were selected for the purpose of this study. The main characteristics can be found in Table 1.

**Table 1:** The selected foods with their (weighted) percentage of never-consumers and daily consumers, Belgian National Food Consumption Survey 2004

	Percentage of never-consumers	Weighted percentage of never-consumers	Daily consumers
Water	1.9%	1.8%	79.0%
Cheese	6.7%	4.6%	23.3%
Fat Spread	31.7%	29.1%	39.3%

The BNFCS2004 made use of stratified sampling. To make the sample representative for the whole Belgian population (between 15 and 74 years) a weighting factor was calculated to compensate for the unequal sampling probability. The weighting factor can be used to convert the percentage of never-consumers towards the weighted percentage of never-consumers

Table 1 shows that all food items fulfill the predefined requirements. Firstly the proportion of never-consumers and the weighted proportion of never-consumers is different for the selected food items. Secondly the proportion of daily consumers for all food items is sufficiently large to avoid convergence problems in SPADE [5, 21, 22].

### Evaluation of the simulation

In order to double check the simulation process, the usual intake distribution of the simulated BNFCS2004 and the simulated “true”, both for the *consumers only*, were compared with the results obtained from the original BNFCS2004 for the consumers only. The estimated usual intake distributions for the water, the cheese and the fat spread dataset for the consumers-only are shown in Table 2A-C, for one of the four simulations.

**Table 2:** Usual intake distribution (g/day) for *consumers only* in the different age strata, Belgian National Food Consumption Survey 2004

A. Water									
	${\sigma}_{b, t}^2$	${\sigma}_{w, t}^2$	*σ* ^2^	mean	P0.05	P0.25	P0.5	P0.75	P0.95
15–18 year									
Original BNFCS2004	3.4	4.7	8.1	600	81	330	547	808	1304
Simulated BNFCS2004	2.9	4.0	6.9	601	69	329	553	807	1302
Simulated “True”	/	/	3.3	595	82	324	540	807	1282
19–59 year									
Original BNFCS2004	7.6	6.2	13.8	712	99	372	630	960	1596
Simulated BNFCS2004	10.2	8.5	18.7	668	91	336	585	905	1528
Simulated “True”	/	/	7.5	711	94	367	630	961	1581
60–74 year									
Original BNFCS2004	9.5	5.3	14.8	610	75	286	515	828	1474
Simulated BNFCS2004	8.0	4.1	12.1	602	73	280	504	815	1467
Simulated “True”	/	/	9.6	608	71	281	510	825	1458
B. Cheese									
15–18 year									
Original BNFCS2004	0.40	1.61	2.01	30	9	19	28	39	58
Simulated BNFCS2004	0.33	1.74	2.07	29	10	19	27	37	54
Simulated “True”	/	/	0.40	30	8	18	28	39	59
19–59 year									
Original BNFCS2004	0.26	0.93	1.19	34	8	21	32	45	68
Simulated BNFCS2004	0.40	1.22	1.62	34	7	20	32	45	69
Simulated “True”	/	/	0.26	35	8	21	33	46	70
60–74 year									
Original BNFCS2004	0.23	0.33	0.56	29	5	15	25	38	65
Simulated BNFCS2004	0.21	0.28	0.49	28	6	14	24	37	63
Simulated “True”	/	/	0.23	28	5	14	25	38	64
C. Fat Spread									
15–18 year									
Original BNFCS2004	0.72	0.99	1.71	12	2	5	9	15	31
Simulated BNFCS2004	1.22	0.68	1.90	11	1	4	7	14	32
Simulated “True”	/	/	0.71	12	2	5	9	15	30
19-59 year									
BNFCS2004	1.38	1.16	2.54	20	2	7	13	25	58
Simulated BNFCS2004	1.51	1.18	2.69	20	3	7	14	26	58
Simulated “True”	/	/	1.49	20	2	7	14	25	60
60–74 year									
BNFCS2004	3.03	2.18	5.21	33	5	14	26	44	84
Simulated BNFCS2004	2.67	2.27	4.94	35	6	16	28	46	85
Simulated “True”	/	/	3.02	33	4	14	26	45	85

*Abbreviations*: *original BNFCS2004* the usual intake distribution obtained from the original BNFCS2004 data, *simulated BNFCS2004* the usual intake distribution obtained from the simulated BNFCS2004 data, *simulated “true”* the usual intake distribution obtained from the simulated “true” intake data. The table also shows the residual variance (*σ* ^2^) and the division of this variance in the within (${\sigma}_{w, t}^2$)- and between (${\sigma}_{b, t}^2$)-individual variance, these variances were estimated on the transformed scale

The usual intake distributions (g/day) for *consumers only* obtained by the original BNFCS2004, the simulated BNFCS2004 and the simulated “true” are shown in Table 2A-C. The mean and median of the usual intake distributions are very similar for all three food items. However, the differences in the usual intake distributions for consumers only becomes larger in the more extreme percentiles. Probably this is caused by the difference in the within- and between-individual variance in the original BNFCS2004 and the simulated BNFCS2004. Meanwhile the between-individual variance is similar in all age strata for the original BNFCS2004 and the simulated “true”, which is a consequence of the method used to simulate the simulated “true” usual intake distribution.

### Effect of the inclusion of FFQ information during modeling

After adding the correct proportion of never-consumers in each of the age strata, the three age-strata of the water, cheese and fat spread dataset were combined. The usual intake distribution for *the whole population* (both (non-)consumers and never-consumers) was obtained by modeling the simulated BNFCS2004 with the SPADE 2-part model, once with and once without the inclusion of the FFQ information on the never-consumers. The obtained usual intake distributions for the whole Belgian population (15–74 years), together with the relative difference as compared to the simulated “true” usual intake distribution are shown in Table 3A-C for water, cheese and fat spread for one of the four simulations.

**Table 3:** Usual intake distribution (g/day) for the *whole (consumers and never-consumers)* Belgian population (15–74 years), after weighting, Belgian National Food Consumption Survey 2004

A. Water							
	P0.025	P0.05	P0.25	P0.5	P0.75	P0.95	P0.975
Simulated BNFCS2004 Without FFQ information	27	60	283	515	817	1420	1667
Simulated BNFCS2004 With FFQ information	10	51	280	508	811	1423	1678
Simulated “true”	11	58	335	593	921	1535	1778
Without/“true”	2.45	1.03	0.84	0.87	0.89	0.93	0.94
With/“true”	0.91	0.88	0.84	0.86	0.88	0.93	0.94
B. Cheese							
Simulated BNFCS2004 Without FFQ information	2	4	15	26	40	67	78
Simulated BNFCS2004 With FFQ information	0	1	16	28	42	70	82
Simulated “true”	0	2	18	30	44	67	77
Without/“true”	/	2	0.83	0.87	0.91	1.00	1.01
With/“true”	/	0.5	0.89	0.93	0.95	1.04	1.06
C. Fat spread							
Simulated BNFCS2004 Without FFQ information	0	0	2	8	21	56	75
Simulated BNFCS2004 With FFQ information	0	0	0	8	20	51	67
Simulated “true”	0	0	0	8	22	58	75
Without/“true”	/	/	/	1	0.95	0.97	1
With/“true”	/	/	/	1	0.91	0.88	0.89

*Abbreviations*: *without FFQ information* usual intake distribution, distribution of the simulated BNFCS2004; without inclusion of FFQ information on the never consumers, *with FFQ information* the same but after inclusion of the information on the never consumers; *simulated “true”* simulated “true” usual intake distribution, *without/“true”* the ratio of without FFQ information to simulated “true”, *with/“true”* the ratio of with FFQ information to simulated “true”

The usual intake distributions for *the whole population* obtained after SPADE modeling (with and without FFQ information) are similar to the simulated “true” usual intake distribution for cheese and fat spread. The absolute values are somewhat different for water, however the relative differences are not that large and are similar to those found in the cheese dataset (Table 3A-C). For all three foods the largest difference in the usual intake distributions between the SPADE model without versus with FFQ information was observed for the lower percentiles. Taking into account the correct proportion of never-consumers resulted in a downwards correction of the usual intake at the lower percentiles. In addition there could be estimated correctly that the proportion of never-consumers was higher than 2.5% for cheese and higher than 25% for fat spread. The influence of the inclusion of the information on the never-consumers while estimating the median and the higher percentiles seemed to be limited for all three food items.

**Fig. 5:**
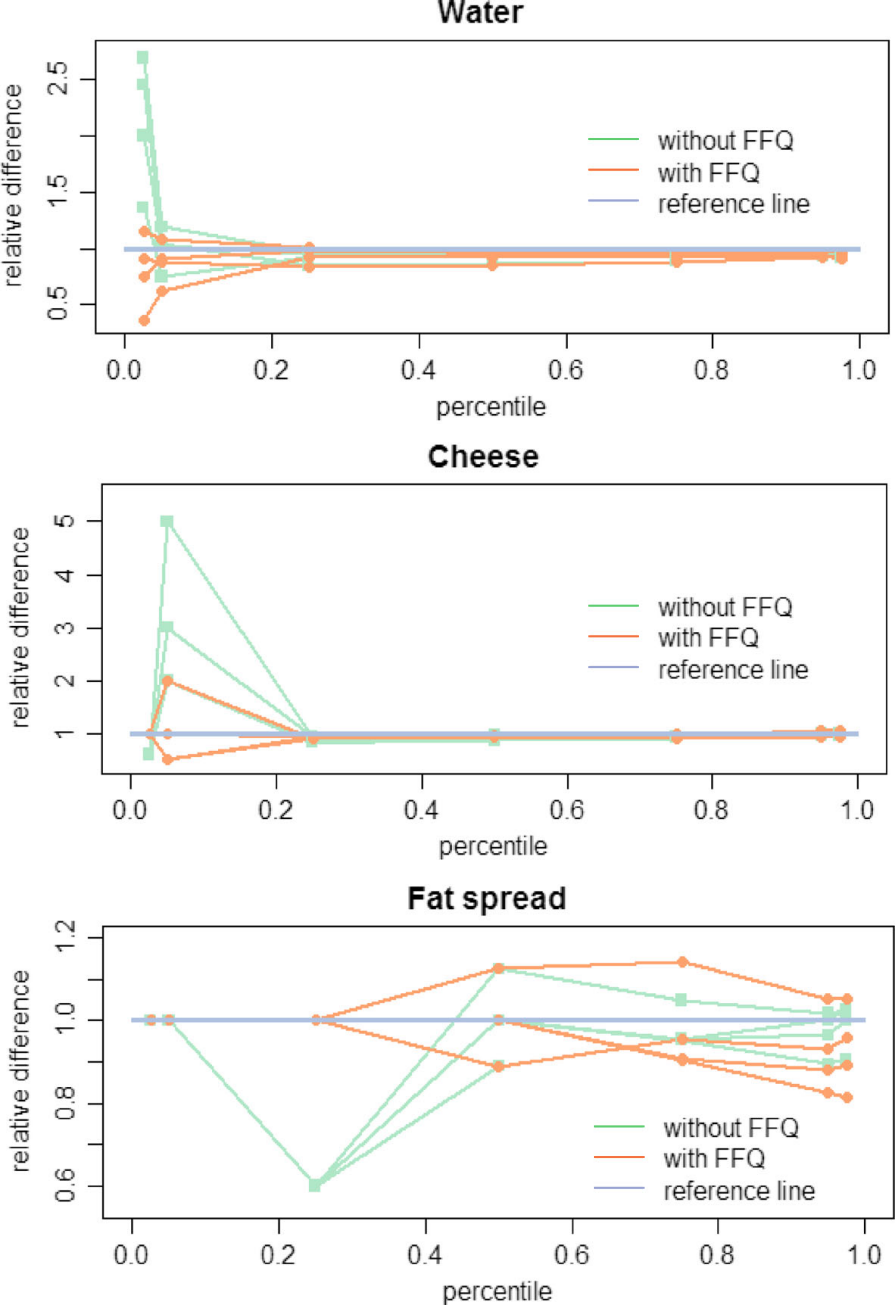
Relative fit of the SPADE 2-part model without/with inclusion of FFQ information on never-consumers, Belgian National Food Consumption Survey 2004. Legend: Relative differences of the usual intakes in function of the percentiles for four replicate simulations. *Without FFQ* presents the ratio of the usual intake amount (g/day) obtained with the SPADE 2-part model without FFQ information on never-consumers, divided by the simulated “true” usual intake amount (g/day). *With FFQ* the same, but with the inclusion of the FFQ information on never-consumers. The reference line represents a ratio of one, a ratio equal to one indicates that the model fitted by the SPADE 2-part model gives exactly the same result as the simulated “true” usual intake distribution

The relative difference of the usual intake distribution without and with inclusion of FFQ information obtained from the simulated BNFCS2004, to the simulated “true” usual intake distribution for the **whole population** were plotted in function of the corresponding percentiles. Figure 5 shows the results of the simulation together with three replicate simulations to get an idea of the variability of the simulations for the water, cheese and fat spread dataset.

Figure 5 confirms the observations from Table 3A-C. When the FFQ information on never-consumers is used we found that (a) the proportion of never-consumers can be estimated more correctly and (b) the usual intake at the lower percentiles obtained a downwards correction. The benefits of the inclusion of information on the never-consumers, already shows up when the proportion of never-consumers is low (e.g. the water dataset). However, the benefits from the inclusion of the information on the never-consumers increases as the number of never-consumers increases.

To allow the within simulation comparison of the effect of the inclusion of FFQ information, the Additional file 2 contains one figure per simulation.

## Discussion

The inclusion of FFQ information for the estimation of the usual intake distribution is possible in two different ways: (a) use of FFQ information as a covariate or (b) use of the basic FFQ information to identify the correct proportion of never-consumers [6]. The main goal when using the FFQ information as a covariate is to improve the estimation of the intake frequency [6, 7, 16, 18, 19], whereas inclusion of the FFQ information to identify the never-consumers allows for reflecting the correct proportion of never-consumers in the population [6, 16, 19]. This study focused on the second option. Goedhart et al. [6] performed a large simulation study and studied amongst others the effect of the use of FFQ information to identify the never-consumers. The current study is somehow similar, but using the SPADE method only. In addition the simulations in this study were based on real food items that were assessed in the Belgian population, whereas in Goedhart et al. [6] the usual intake data were artificial.

### Evaluation of the simulation

The mean and the median for **the consumers only** of the simulated BNFCS2004 and the simulated “true” are similar to those in the original BNFCS2004. The within- and between- individual variance are different in the original BNFCS2004 and the simulated BNFCS2004. A possible explanation of such a difference could be the small number of simulated cases, e.g. for the water dataset 745 adolescents, 807 adults and 766 elderly. However, as expected, the between-individual variance in the original BNFCS2004 and the variance of the simulated “true” usual intake are similar [6, 8]. The SPADE 2-part model namely states to estimate the between- and within-individual variance, since the aim is to estimate the usual intake distribution of the population, SPADE will remove the within-individual variance from the usual intake distribution [21, 23]. As a consequence the variance of the original BNFCS2004 usual intake distribution will be equal to the between-individual variance. Simulating 1000 recall days for each individual in the simulated “true” database corresponds to following these individuals during 2 years and 8 months. When an individual is followed for so many days, the usual intake of that individual is more certain, and (almost) no within-individual variance will be left [6, 8]. The variance in the simulated “true” usual intake is indeed nearly equal to the between-individual variance observed in the different age strata in the original BNFCS2004, as shown in Table 2A-C.

Since the interest lays in estimating the usual intake at population level, rather than the individual intake, the goal is to integrate out the within-individual variance from the data, to obtain a usual intake distribution where only the between-individual variance is considered [5]. This approach assumes that the mean of a sufficient amount of 24HRs in one individual results in the “true” usual intake of that individual. This implicates the assumption that the 24HR is unbiased at the individual level [7, 8]. However, biomarker studies of dietary intake showed that self-report instruments are biased [24–27].

A limitation during modeling was related to the fact that the simulation was performed in the three age strata separately. As a consequence the whole population simulated “true” usual intake distribution consists of three fitted models, more precisely one model in each age stratum. At first sight the same is happening in the simulated BNFCS2004, but the difference is that the data are remodeled by the SPADE 2-part model, after merging the three age strata together. At this point only one model is fitted for the complete age range and this affects the usual intake distribution. For instance the water dataset: when working in the different age strata (three models) it was shown that the water consumption was highest in the adults age group (534 g/day in adolescent, 619 g/day in adults and 499 g/day in elderly). However, when the SPADE 2-part model (only one model) was used on the simulated BNFCS2004 data, the intake amount seemed to decrease with the age from 539 g/day in adolescents to 525 g/day in adults and 428 g/day in elderly. In addition the adults age group was underrepresented most and received the highest weight [28]. All this together resulted in a higher usual intake of water in the simulated “true” usual intake distribution and an underestimation of the intake of water when the SPADE 2-part model was used on the simulated BNFCS2004.

Another observation is that in the fat spread dataset the SPADE 2-part model without FFQ information on never-consumers (which does not take into account the proportion of never-consumers) predicts zero intakes for both P0.025 and P0.05. There are two possible explanations for those results: (a) because of the larger proportion of days without intakes, intake amounts will be regularly multiplied with an intake frequency close to zero, (b) at the same time fat spreads are consumed in rather small quantities. In this situation the benefit of the inclusion of FFQ information is no longer present in the lowest percentiles (e.g. P0.025 and P0.05 in the fat spread dataset). However, benefits were still present in the percentiles just above (e.g. P0.25 in the fat spread dataset).

### Effect of the inclusion of FFQ information during modeling

Inclusion of FFQ information to identify the never-consumers is not beneficial while estimating the higher percentiles of the usual intake distribution. On the other hand the results indicate that using the FFQ data to identify the never-consumers is crucial while estimating the lower percentiles of the usual intake distribution, even when the proportion of never-consumers is low. E.g. a benefit was seen for water where only 2% indicated to be a never-consumer. Both results were in accordance with the findings in the simulation study of Goedhart et al. [6].

This means in practice that when interest is in the food safety issue, the goal is typically to focus on the consumers with the highest intake, as the high consumers are at risk [6, 29]. Since inclusion of FFQ information on the never-consumers does not seem to improve the estimation of the higher percentiles, inclusion of this information will probably have no benefits.

On the other hand if interest is in the food adequacy issue, the interest is typically in the individuals with the lowest intake [29]. Since inclusion of information on never-consumers improves the estimation of the lowest percentiles, inclusion of the information on the never-consumers will be beneficial. In a national food consumption survey the usual intake distribution of the whole population is measured, both upper and lower percentiles are of interest in this situation [6, 29]. Again inclusion of the FFQ information on the never-consumers will be beneficial to better estimate the lower percentiles of the usual intake distribution. Finally the benefit of inclusion of FFQ information on never-consumers to estimate the lower percentiles of the usual intake distribution becomes larger, as the proportion of never-consumers increases.

### Strength and limitations of the study

The simulation was performed in the three age strata separately, with the consequence described above. In addition, this age stratification also limited the number of food items that could be selected. Namely, the number of individuals with consumption on both recall days had to be sufficiently large in all subgroups to avoid convergence problems in SPADE [5, 21, 22]. Though this problem is not unique for SPADE, also other statistical modeling methods, like the ISU (Iowa State University) and the NCI (National Cancer Institute) method require a sufficient number of respondents with at least two positive intake days in order to avoid convergence problems [7, 19, 30]. The decision to perform this simulation in the three separate age strata was made because usual intakes can vary substantially depending on the age of the individuals [7, 8, 31]. This was also shown in the results section, especially for fat spread and water. When all age groups would have been simulated at the same time, the differences over the age groups would no longer be present in the same magnitude. Since SPADE can take into account the age during modeling, a part of the age effect would still be captured [21, 22].

The added value of the current study is that the simulation was performed on the basis of real data, which allows a better evaluation of the effect of the inclusion of information on never-consumers in a real life situation. In addition, in the current simulation some difficulties were encountered in the translation from theory to practice. Firstly, it is not always easy to make a straightforward link between the FFQ questions and the food items obtained from the 24HR. This illustrates at the same time the importance of constructing the FFQ questions in function of the analysis that are planned. Secondly, the use of real data showed convergence problems, when the number of respondents with two positive intakes during the recall days became too low, as was shown in other studies [7, 16, 19, 30]. Such convergence problems occur more often during subgroup analysis, because of the smaller number of observations. These kinds of problems are more difficult to spot when the simulation is purely theoretical.

## Conclusions

The inclusion of FFQ information to identify the never-consumers improves the estimation of the usual intake distribution, but only at the lower percentiles. When interest is in the whole usual intake distribution (lower and upper percentiles) or interest is only in the lower percentiles of the usual intake distribution, inclusion of this FFQ information is beneficial even when the proportion of never-consumers is low. However, when interest lies only in the higher percentiles of the usual intake distribution, inclusion of FFQ information on the never-consumers will have no benefit.

## Additional files

Additional file 1: Details on SPADE and the simulation process. (DOCX 253 kb)

Additional file 2: Relative fit of the SPADE 2-part model without/with inclusion of FFQ information on never-consumers, Belgian National Food Consumption Survey 2004. Legend: Relative differences of the usual intakes in function of the percentiles for the four replicate simulations separately. Without FFQ presents the ratio of the usual intake amount (g/day) obtained with the SPADE 2-part model without FFQ information on never-consumers, divided by the simulated “true” usual intake amount (g/day). With FFQ the same, but with the inclusion of the FFQ information on never-consumers. The reference line represents a ratio of one, which indicates that the model fitted by the SPADE 2-part model gives exactly the same result as the simulated “true” usual intake distribution. (PDF 1529 kb)

## Abbreviations

24HR: 24-hour recall
BNFCS2004: Belgian National Food Consumption Survey 2004
EFCOSUM: European Food Consumption Survey Method project
EPIC-Soft: European Prospective Investigation into Cancer and Nutrition software
FFQ: Food frequency questionnaire
ISU: Iowa State University method
NCI: National Cancer Institute method
SPADE: Statistical Program for Dietary Exposure
λ_bc_: Box-Cox transformation parameter
μ: Mean usual intake
σ_b_: Between-individual standard deviation
σ_w_: Within-individual standard deviation

## References

[1] De Vriese S, De Backer G, De Henauw S, Huybrechts I, Kornitzer K, Leveque A (2005). The Belgian food consumption survey: aims, design and methods. Arch Public Health.

[2] WIV-ISP. More information objectives. 2014. https://fcs.wiv-isp.be/info/SitePages/Objectives.aspx?WikiPageMode=Edit&InitialTabId=Ribbon.EditingTools.CPEditTab&VisibilityContext=WSSWikiPage. Accessed 26 Aug 2016.

[3] Ezzati M, Riboli E (2013). Behavioral and dietary risk factors for noncommunicable diseases. N Engl J Med.

[4] Brussaard J, Johansson L, Kearney J (2002). Rationale and methods of the EFCOSUM project. Eur J Clin Nutr.

[5] Dodd KW, Guenther PM, Freedman LS, Subar AF, Kipnis V, Midthune D (2006). Statistical methods for estimating usual intake of nutrients and foods: a review of the theory. J Am Diet Assoc.

[6] Goedhart PW, van der Voet H, Knüppel S, Dekkers ALM, Dodd KW, Boeing H, et al. A comparision by simulation of different methods to estimate the usual intake distribution for episodically consumed foods 2012. Supporting publications 2012: En299. www.efsa.europa.eu/publications. Accessed 26 Aug 2016.

[7] Tooze JA, Midthune D, Dodd KW, Freedman LS, Krebs-Smith SM, Subar AF (2006). A new statistical method for estimating the usual intake of episodically consumed foods with application to their distribution. J Am Diet Assoc.

[8] Tooze JA, Kipnis V, Buckman DW, Carroll RJ, Freedman LS, Guenther PM (2010). A mixed-effects model approach for estimating the distribution of usual intake of nutrients: the NCI method. Stat Med.

[9] Souverein OW, Dekkers AL, Geelen A, Haubrock J, de Vries JH, Ocke MC (2011). Comparing four methods to estimate usual intake distributions. Eur J Clin Nutr.

[10] Brussaard J, Löwik M, Steingrimsdottir L, Møller A, Kearney J, De Henauw S (2002). A European food consumption survey method--conclusions and recommendations. Eur J Clin Nutr.

[11] Beaton GH, Milner J, Corey P, McGuire V, Cousins M, Stewart E (1979). Sources of variance in 24-hour dietary recall data: implications for nutrition study design and interpretation. Am J Clin Nutr.

[12] Beaton GH, Milner J, McGuire V, Feather T, Little JA (1983). Source of variance in 24-hour dietary recall data: implications for nutrition study design and interpretation. Carbohydrate sources, vitamins, and minerals. Am J Clin Nutr.

[13] Mackerras D, Rutishauser I (2005). 24-hour national dietary survey data: how do we interpret them most effectively?. Public Health Nutr.

[14] National Research Council, Subcommittee on Criteria for Dietary Evaluation. Nutrient adequacy: assessment using food consumption surveys. Washington: DC: National Academy Press; 1986. 25032431

[15] Nusser SM, Carriquiry AL, Dodd KW, Fuller WA (1996). A semiparametric transformation approach to estimating usual daily intake distributions. J Am Stat Assoc.

[16] Haubrock J, Nothlings U, Volatier JL, Dekkers A, Ocke M, Harttig U (2011). Estimating usual food intake distributions by using the multiple source method in the EPIC-Potsdam calibration study. J Nutr.

[17] Slob W (2006). Probabilistic dietary exposure assessment taking into account variability in both amount and frequency of consumption. Food Chem Toxicol.

[18] Subar AF, Dodd KW, Guenther PM, Kipnis V, Midthune D, McDowell M (2006). The food propensity questionnaire: concept, development, and validation for use as a covariate in a model to estimate usual food intake. J Am Diet Assoc.

[19] Kipnis V, Midthune D, Buckman DW, Dodd KW, Guenther PM, Krebs-Smith SM (2009). Modeling data with excess zeros and measurement error: application to evaluating relationships between episodically consumed foods and health outcomes. Biometrics.

[20] Slimani N, Valsta L (2002). Perspectives of using the EPIC-SOFT programme in the context of pan-European nutritional monitoring surveys: methodological and practical implications. Eur J Clin Nutr.

[21] Dekkers AL, Verkaik-Kloosterman J, van Rossum CT, Ocké MC (2014). SPADE, a new statistical program to estimate habitual dietary intake from multiple food sources and dietary supplements. J Nutr.

[22] Dekkers AL, Verkaik-Kloosterman J, van Rossum CT, Ocké MC. SPADE: Statistical Program to Asses habitual Dietary Exposure, User’s Manual version 2.0, for SPADE version 3.0; December 2014. Bilthoven: RIVM (National Institute for Public Health and the Environment); 2014.

[23] Dekkers ALM, Slob W (2012). Gaussian Quadrature is an efficient method for the back-transformation in estimating the usual intake distribution when assessing dietary exposure. Food Chem Toxicol.

[24] Freedman LS, Midthune D, Carroll RJ, Krebs-Smith S, Subar AF, Troiano RP (2004). Adjustments to improve the estimation of usual dietary intake distributions in the population. J Nutr.

[25] Macdiarmid J, Blundell J (1998). Assessing dietary intake: who, what and why of under-reporting. Nutr Res Rev.

[26] Subar AF, Kipnis V, Troiano RP, Midthune D, Schoeller DA, Bingham S (2003). Using intake biomarkers to evaluate the extent of dietary misreporting in a large sample of adults: the OPEN study. Am J Epidemiol.

[27] Kipnis V, Subar AF, Midthune D, Freedman LS, Ballard-Barbash R, Troiano RP (2003). Structure of dietary measurement error: results of the OPEN biomarker study. Am J Epidemiol.

[28] Hahs-Vaughn DL (2005). A primer for using and understanding weights with national datasets. J Exp Educ.

[29] De Boer E, Slimani N, van’t Veer P, Boeing H, Feinberg M, Leclercq C (2011). The European food consumption validation project: conclusions and recommendations. Eur J Clin Nutr.

[30] Nusser SM, Fuller WA, Guenther PM, Lyberg L, Biemer P, Collins M, DeLeeuw E, Dippo C, Schwartz N (1997). Estimating usual dietary intake distributions: adjusting for measurement error and nonnormality in 24-hour food intake data. Survey measurement and process quality.

[31] Waijers P, Dekkers ALM, Boer JMA, Boshuizen HC, van Rossum CTM (2006). The potential of AGE MODE, an age-dependent model, to estimate usual intakes and prevalences of inadequate intakes in a population. J Nutr.

