# Supplementary material for: The added value of food frequency questionnaire (FFQ) information to estimate the usual food intake based on repeated 24-hour recalls
Source: Arch Public Health. 2017 Oct 30;75:46. doi: 10.1186/s13690-017-0214-8 (PMC5662101; doi:10.1186/s13690-017-0214-8)
Supplement: Supplementary file 1 — Details on SPADE and the simulation process. (DOCX 253 kb) [file 13690_2017_214_MOESM1_ESM.docx]

**Appendix: Details on SPADE and the simulation process**

Statistical program to assess dietary exposure

**Figure 1:** Basic idea of the SPADE 2-part models.


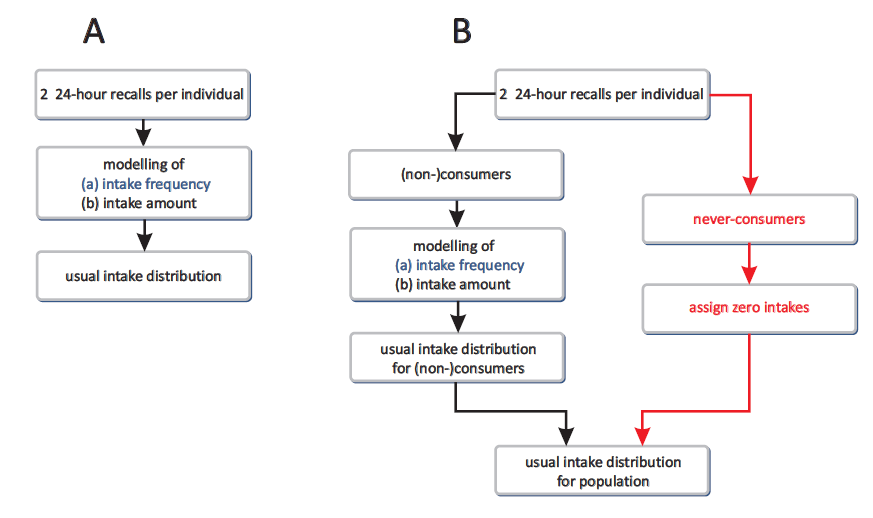


The panel on the left (A) shows the SPADE 2-part model without the inclusion of the information on the never-consumers. The panel on the right (B) shows the SPADE 2-part model with the inclusion of the information on the never-consumers. Note that based on the FFQ information the population is divided into the consumers and non-consumers on one hand; and the never-consumers on the other hand [1-2].

The panel on the left in figure 1 shows the basic steps of the SPADE 2-part model without inclusion of the never-consumers information: 24HRs of all respondents are used to model (a) the intake frequency and (b) the intake amount. Combining both results in the usual intake distribution for whole the population [1-2].

The panel on the right in figure 1 presents the basics steps of the SPADE 2-part model with inclusion of the never-consumers information. By using the FFQ information on the never-consumers the model divides the population in (a) the consumers and the non-consumers, and (b) the never-consumers. The usual intake distribution of the (non-) consumers is modeled in the same way as described above, however it will now result in the usual intake distribution for the consumers only. The never-consumers all get assigned a zero usual intake. Finally, the usual intake distribution of the (non-) consumers and the zero intakes of the never-consumers are combined to obtain the usual intake distribution for whole the population, which will reflect the correct proportion of never-consumers [1-2].

Figure 2 shows in detail how SPADE models the usual intake distribution. The first step of the SPADE 2-part model estimates the consumption frequency. Three situations are possible: 2 days, 1 day or no days with consumption of the specific food item. The second part of SPADE models the consumption amounts on consumption days. For the amounts modeling purpose, only the subjects with at least one positive intake (during the two days of recall) are taken into account. The positive intakes of all subjects with one positive intake, and randomly one positive intake from the subjects with two positive intakes are selected. The selected intakes are transformed to normality using a Box-Cox transformation. These transformed amounts are then modeled as a function of age by a fractional polynomial regression and all model parameters are estimated including the total residual variance. Then the within- and between-individual variances are estimated, using the data of subjects with two non-zero intakes. Next the ratio of the latter between- and within-individual variance is applied to the total residual variance in order to estimate the total between- and within-individual variance. A Gaussian quadrature back-transformation is subsequently used to (a) integrate out the within-individual variance and (b) to back-transform the resulting shrunken distribution to the original scale [1-3].

In the third step the distributions of the intake frequency and intake amount are combined by a Monte Carlo simulation to obtain the usual intake distribution [1-2].

**Figure 2:** Detail of the intake frequency and the intake amount modeling in the SPADE 2-part model.


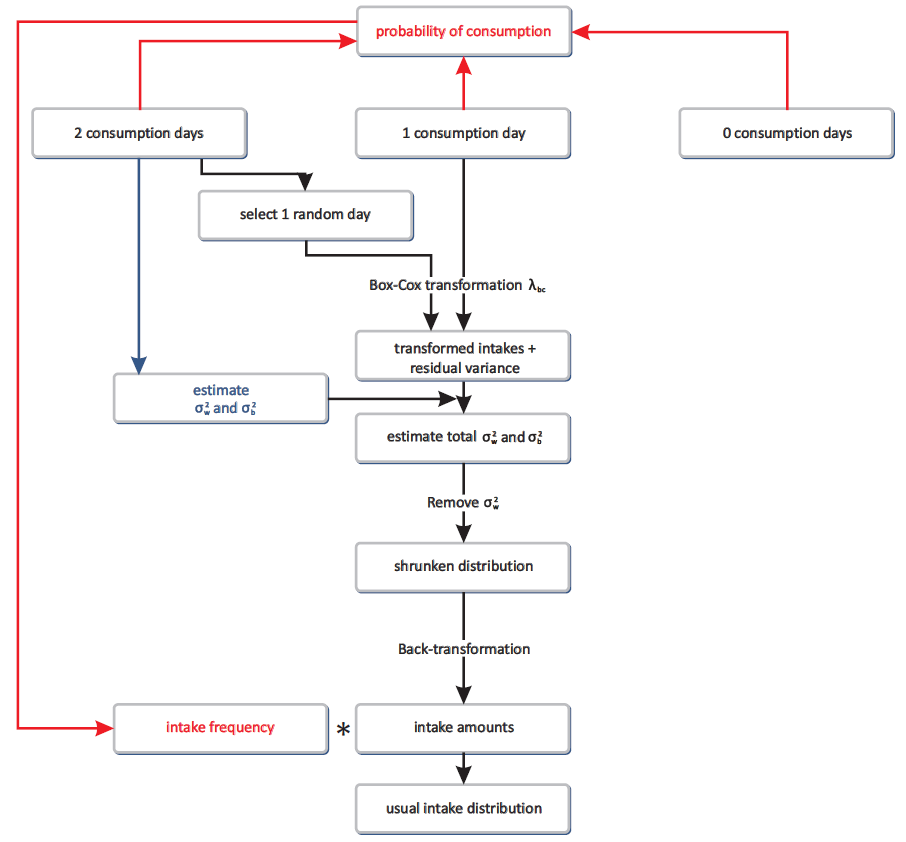


Firstly, the SPADE 2-part model estimates the intake frequency and then it estimates the intake amounts. Finally, the intake frequency and the intake amount are combined to obtain the usual intake distribution of (a) whole the population when information on the never-consumers is not taken into account; or (b) the consumers only when information on the never-consumers is taken into account [1-2].

Selection of the food items

We used the following criteria to select the food items used in the current study: (a) they needed to have different proportions of never-consumers, and (b) even when the proportion of never-consumers was large, the amount of participants consuming the food on both recall days had to be sufficiently large, to avoid convergence problems in SPADE (convergence problems occur when the available amount of data is insufficient to obtain an adequate model fit) [1-2, 4].

Data simulation

***The simulation of a new database***

The simulated BNFCS2004 was generated by simulating two 24HRs and basic FFQ information (only information on never-consumers versus consumers). The simulated BNFCS2004 was limited to individuals, aged 15-74 years (n=2363). The simulation was performed stratified in the three different age groups (15-18 years, 19-59 years and 60-74 years), which allows for more variation of the food consumption in function of age. The simulation took place in two stages: (a) simulate the consumers only and (b) simulate the never-consumers only (never-consumers are individuals who indicated in the FFQ that they never consumed the food item during the last 12 months).

*Simulation of the consumers only – Simulated BNFCS2004 consumers only*

For the simulation of the consumers, an approach similar to Souverein et al. [5] was used. SPADE models both the intake frequency and the intake amounts were only modeled in function of age [1-2]. To avoid convergence problems only the intake amounts were modeled in function of age during the simulation.

First all never-consumers were excluded from the original BNFCS2004 database using FFQ data, resulting in a subdatabase with consumers only. Then the SPADE 2-part model without information on the never-consumers was used to obtain the usual intake distribution for consumers only from the original BNFCS2004. During the modeling some parameters were extracted: the mean usual intake for every age (μ_age_), the within-individual standard deviation (σ_w_), the between-individual standard deviation (σ_b_) and the Box-Cox transformation parameter (λ_bc_) (figure 3 box A).

After the extraction of all needed parameters the simulation could start on the transformed scale. Firstly the age for all respondents was simulated, making the assumption that the age was uniformly distributed in each of the three age strata. Then each respondents' mean usual intake was simulated, using a normal distribution, with the mean equal to the age dependent mean usual intake and with the variance equal to the between-individual variance. Next two 24HRs were simulated for each respondent using again a normal distribution with the mean equal to the individuals mean usual intake (simulated in the previous step) and the variance equal to the within-individuals variance. The within-individual variance was assumed to be equal for each individual. These intakes were then back-transformed to the original scale using λ_bc_.

During this simulation it must be taken into account that consumers don't eat the specific food items every day. Thus also the correct proportion of consumers that consume on any-, on one- or on both days had to be taken into account. The intake frequency was simulated using a beta-binomial model, which considered the mean intake frequency and the correlation of the intake frequencies. This model resulted in a yes/no answer on whether the food was consumed on a given day. Since two recall days were considered the model has three possible outcomes: one, two or no days with a positive intake. In the first scenario with two positive intakes both simulated 24HRs were kept. When there was one day with a positive intake, one of the previous simulated intakes was set to zero and finally, both simulated 24HRs were set to zero if there was no day with a positive intake (figure 3 box B).

**Figure 3:** Simulation of the simulated BNFCS2004.


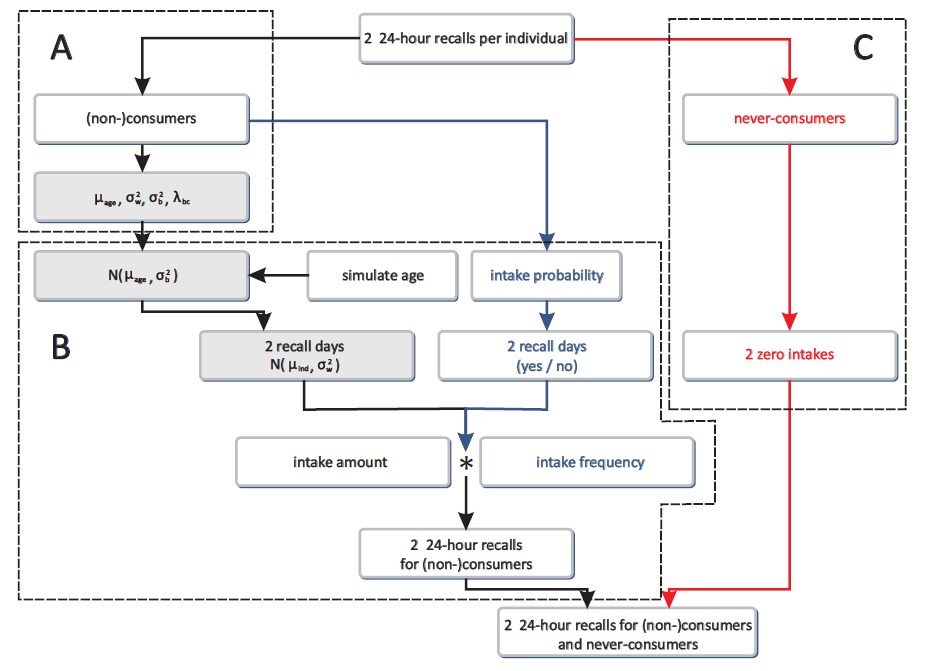


The “simulated BNFCS2004” consisting of two 24HRs per individual was simulated separately in the three age strata: the adolescents (15-18 years), the adults (19-59 years) and the elderly (60-74 years). The grey-shaded steps are performed at the transformed scale. In box A, the consumers only in the “original BNFCS2004” are modeled to extract the needed modeling parameters. In box B, these parameters were used to simulate two 24 HRs for every (non-) consumer resulting in the “simulated BNFCS2004 for consumers only”. In box C, the correct proportion of never-consumers gets assigned two 24 HRs with an intake amount equal to zero. Finally, the two 24 HRs of the (non-) consumers and the never-consumers are combined to obtain the “simulated BNFCS2004”.

Finally, there were some variables that had to be created to allow the SPADE analysis afterwards: an ID code, an interview number (one and two corresponding to the recall day), the gender and the basic FFQ answer (consumer versus never-consumer). For the gender the assumption was made that there are equal amounts of males and females in each of the age strata. The FFQ code was set to one to allow the identification of the consumers. The end result was that for each (non-)consumer two 24HRs were simulated, resulting in the “simulated BNFCS2004 consumers only”.

*Simulation of the never-consumers – Simulated BNFCS2004 never-consumers only*

The correct number of never-consumers in each age stratum was calculated based on the FFQ data of the original BNFCS2004. The never-consumers got assigned a zero intake on both recall days. The corresponding FFQ variable received a value of zero to indicate the never-consumers. The simulation of the ID number, the age, the gender and interview number were similar to the consumers-only procedure. Thus for each never-consumer two 24HRs with a consumption equal to zero were generated resulting in the “simulated BNFCS2004 never-consumers only” (figure 3 box C).

***The simulation of a “true” usual intake distribution***

The simulation of the simulated "true" usual intake distribution was very similar and was based on the methods described by Goedhart et al. [6], Tooze et al. [7] and Souverein et al. [5].

*Simulation of the consumers only – Simulated “true” usual intake distribution consumers only*

The simulation was performed separately in the three age strata. In each stratum 5000 consumers were simulated. In comparison with the simulation of the simulated BNFCS2004 consumers only, the simulation of the "true" usual intake distribution had two major differences. Firstly, the mean for the simulated usual intake was the age-specific median at the transformed scale instead of the mean. Since the median is more robust, this simulation will be more correct when the distribution is not perfectly normal after the Box-Cox transformation. Secondly, instead of simulating two 24HRs per individual, one thousand 24HRs were simulated for each individual, which corresponds to following these individuals during 2 years and 8 months. The median intake over these thousand days can be considered as the "true" usual intake on a consumption day for that individual. Since not all days are consumption days, also the mean consumption frequency during these thousand days had to be simulated, again using a beta-binomial model. After back-transformation, the median intake on consumption days and the mean intake frequency was multiplied for all individuals to obtain their “true” usual intake. These "true" usual intakes for all individuals together resulted directly in the simulated "true" usual intake distribution for the consumers only (figure 4, box B). Thus no additional modeling was needed, since by following the individuals during one thousand days, almost no within-individual variance was left.

*Simulation of the never-consumers – Simulated “true” usual intake for never-consumers*

The proportion of never-consumers in the simulated "true" usual intake distribution has to be the same as the proportion of never-consumers in the original BNFCS2004. The usual intake amount and the usual intake frequency of the never-consumers were set to a value of zero, resulting in the simulated “true” usual intake for never-consumers (figure 4 box C).

**Figure 4:** Simulation of the “true” usual intake distribution.


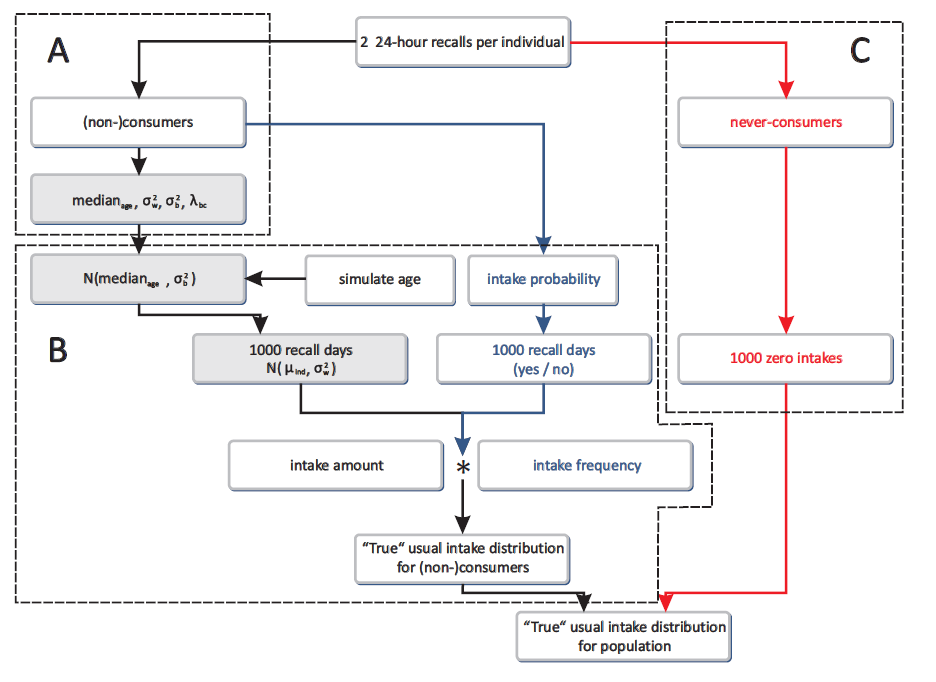
 The “simulated BNFCS2004” consisting of two 24-hour recalls per individual was simulated separately in the three age strata: the adolescents (15-18 years), the adults (19-59 years) and the elderly (60-74 years). The grey-shaded steps are performed at the transformed scale. In box A, the needed modeling parameters were extracted by modeling the original BNFCS2004. In box B, these parameters were used to simulate one thousand 24 HRs for every consumer. The median intake over these one thousand simulated days results in the “true usual intake distribution for consumers only”. In box C, the correct proportion of never-consumers gets assigned one thousand 24 HRs with an intake amount equal to zero, which corresponds to a usual intake of zero. Finally, the usual intake distributions of the consumers and the never-consumers are combined to obtain the “simulated true usual intake distribution”.

**References**

1. Dekkers AL, Verkaik-Kloosterman J, van Rossum CT, Ocké MC. SPADE: Statistical Program to Asses habitual Dietary Exposure, User's Manual version 2.0, for SPADE version 3.0; December 2014. : RIVM2014.

2. Dekkers AL, Verkaik-Kloosterman J, van Rossum CT, Ocké MC. SPADE, a New Statistical Program to Estimate Habitual Dietary Intake from Multiple Food Sources and Dietary Supplements. The Journal of Nutrition. 2014 Dec;144(12):2083-91.

3. Dekkers ALM, Slob W. Gaussian Quadrature is an efficient method for the back-transformation in estimating the usual intake distribution when assessing dietary exposure. Food and Chemical Toxicology. 2012 Oct;50(10):3853-61.

4. Dodd KW, Guenther PM, Freedman LS, Subar AF, Kipnis V, Midthune D, et al. Statistical methods for estimating usual intake of nutrients and foods: A review of the theory. Journal of the American Dietetic Association. 2006 Oct;106(10):1640-50.

5. Souverein OW, Dekkers AL, Geelen A, Haubrock J, de Vries JH, Ocke MC, et al. Comparing four methods to estimate usual intake distributions. European Journal of Clinical Nutrition. 2011 Jul;65:S92-S101.

6. Goedhart PW, van der Voet H, Knüppel S, Dekkers ALM, Dodd KW, Boeing H, et al. A comparision by simulation of different methods to estimate the usual intake distribution for episodically consumed foods2012. Supporting publications 2012: En299. www.efsa.europa.eu/publications

7. Tooze JA, Kipnis V, Buckman DW, Carroll RJ, Freedman LS, Guenther PM, et al. A mixed-effects model approach for estimating the distribution of usual intake of nutrients: The NCI method. Statistics in Medicine. 2010 Nov;29(27):2857-68.
