# Supplementary material for: The added value of food frequency questionnaire (FFQ) information to estimate the usual food intake based on repeated 24-hour recalls
Source: Arch Public Health. 2017 Oct 30;75:46. doi: 10.1186/s13690-017-0214-8 (PMC5662101; doi:10.1186/s13690-017-0214-8)
Supplement: Supplementary file 2 — Relative fit of the SPADE 2-part model without/with inclusion of FFQ information on never-consumers, Belgian National Food Consumption Survey 2004. Legend: Relative differences of the usual intakes in function of the percentiles for the four replicate simulations separately. Without FFQ presents the ratio of the usual intake amount (g/day) obtained with the SPADE 2-part model without FFQ information on never-consumers, divided by the simulated “true” usual intake amount (g/day). With FFQ the same, but with the inclusion of the FFQ information on never-consumers. The reference line represents a ratio of one, which indicates that the model fitted by the SPADE 2-part model gives exactly the same result as the simulated “true” usual intake distribution. (PDF 1529 kb) [file 13690_2017_214_MOESM2_ESM.pdf]

Figure1: Relative fit of the SPADE 2-part model without/with inclusion of FFQ information on never-consumers, Belgian National Food Consumption Survey 2004.

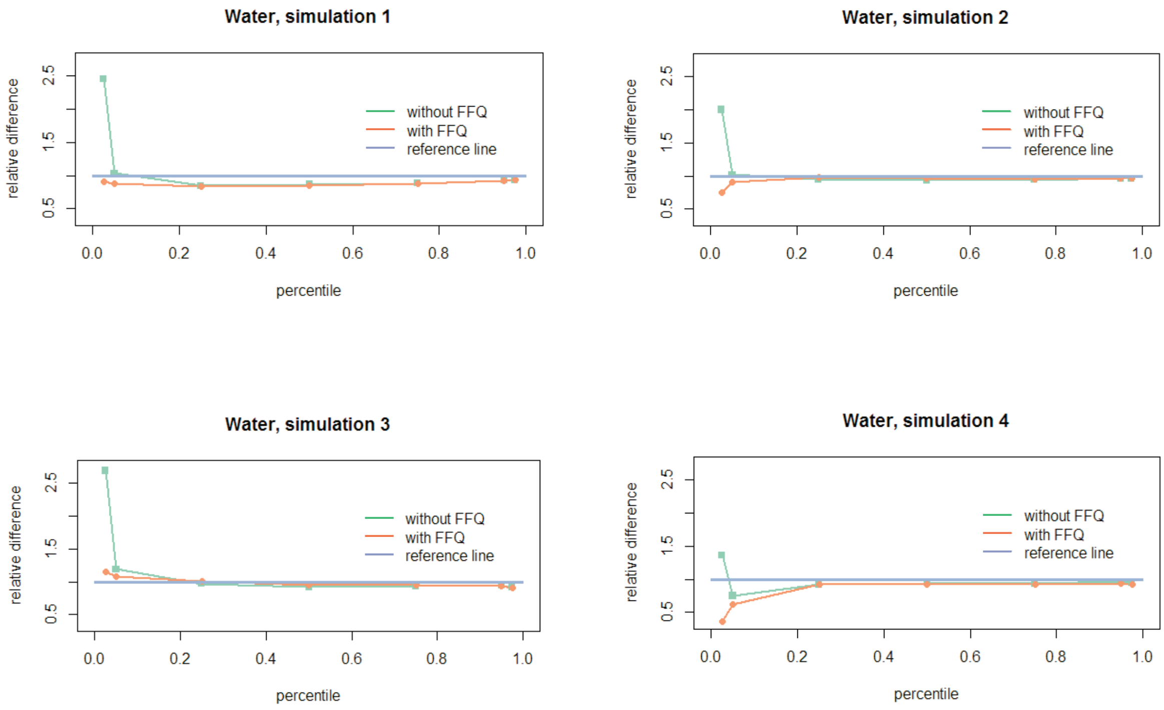

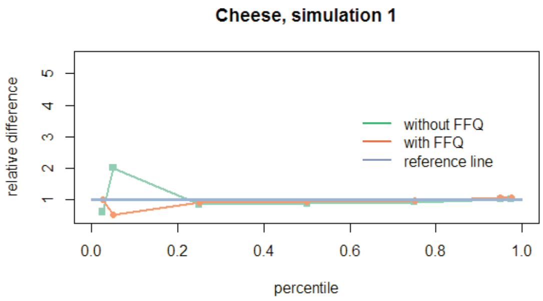
Cheese, simulation 1
relative difference
1
2
3
4
5
0.0
0.2
0.4
0.6
0.8
1.0
percentile
without FFQ
with FFQ
reference line

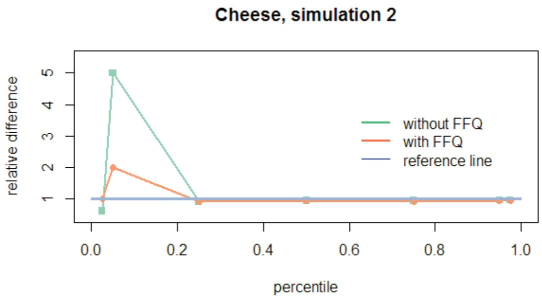
Cheese, simulation 2
relative difference
1
2
3
4
5
0.0
0.2
0.4
0.6
0.8
1.0
percentile
without FFQ
with FFQ
reference line

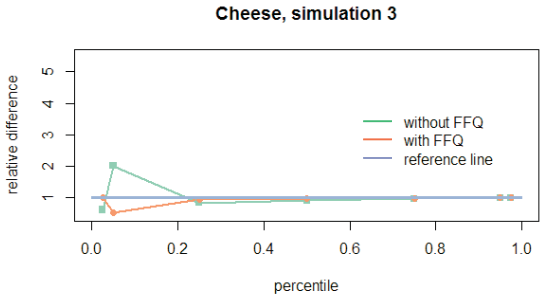
Cheese, simulation 3
relative difference
1
2
3
4
5
0.0
0.2
0.4
0.6
0.8
1.0
percentile
without FFQ
with FFQ
reference line

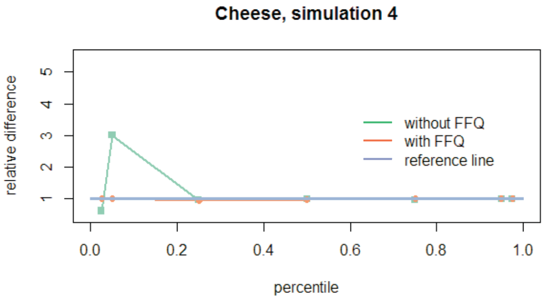
Cheese, simulation 4
relative difference
1
2
3
4
5
0.0
0.2
0.4
0.6
0.8
1.0
percentile
without FFQ
with FFQ
reference line

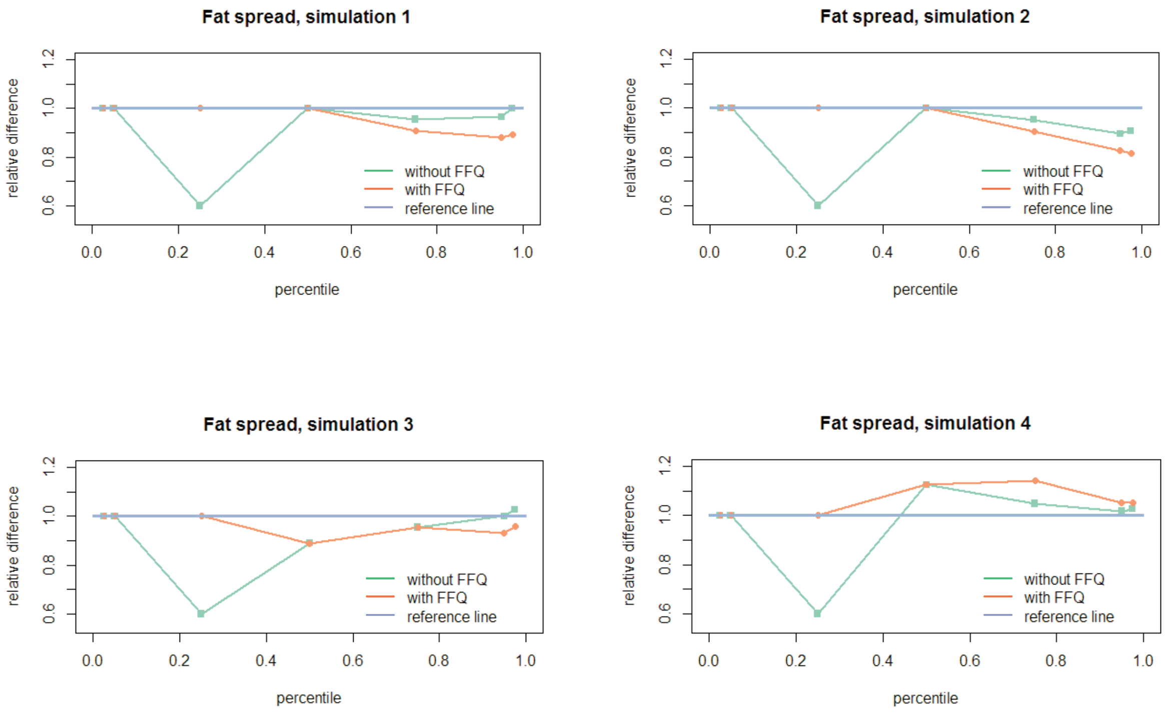

Relative differences of the usual intakes in function of the percentiles for the four replicate simulations separately. *Without FFQ* presents the ratio of the usual intake amount (g/day) obtained with the SPADE 2-part model without FFQ information on never-consumers, divided by the simulated "true" usual intake amount (g/day). *With FFQ* the same, but with the inclusion of the FFQ information on never-consumers. The reference line represents a ratio of one, which indicates that the model fitted by the SPADE 2-part model gives exactly the same result as the simulated "true" usual intake distribution.
